# Effect of Training on Physiological and Biochemical Variables of Soccer Players of Different Age Groups

**DOI:** 10.5812/asjsm.34875

**Published:** 2010-03

**Authors:** Indranil Manna, Gulshan Lal Khanna, Prakash Chandra Dhara

**Affiliations:** 1 Human Performance Lab, Sports Authority of India, J. N. Stadium, New Delhi, India; 2 Department of Health Sciences, Manav Rachana International University, Faridabad, India; 3 Department of Human Physiology, Vidyasagar University, Midnapore, India

**Keywords:** Anaerobic power, VO_2max_, Strength, Body fat, Lipid profile, Soccer

## Abstract

**Purpose:**

To find out the effect of training on selected physiological and biochemical variables of Indian soccer players of different age groups.

**Methods:**

A total of 120 soccer players volunteered for the study, were divided (n = 30) into 4 groups: (i) under 16 years (U16), (ii) under 19 years (U19), (iii) under 23 years (U23), (iv) senior (SR). The training sessions were divided into 2 phases (a) Preparatory Phase (PP, 8 weeks) and (b) Competitive Phase (CP, 4 weeks). The training program consisted of aerobic, anaerobic and skill development, and were completed 4 hrs/day; 5 days/week. Selected physiological and biochemical variables were measured at zero level (baseline data, BD) and at the end of PP and CP.

**Results:**

A significant increase (P < 0.05) in lean body mass (LBM), VO_2max_, anaerobic power, grip and back strength, urea, uric acid and high density lipoprotein cholesterol (HDL-C); and a significant decrease (P < 0.05) in body fat, hemoglobin (Hb), total cholesterol (TC), triglyceride (TG) and low density lipoprotein cholesterol (LDL-C) were detected in some groups in PP and CP phases of the training when compare to BD. However, no significant change was found in body mass and maximal heart rate of the players after the training program.

**Conclusion:**

This study would provide useful information for training and selection of soccer players of different age groups.

## INTRODUCTION

Soccer (football) is unarguably the world's most popular sport. A common aspect of this sport is the necessity of teamwork to complement individual skills. In order to adapt to technical evolution within the game, players have to meet the physical demands required. To achieve the best possible performance, training has to be formulated according to the principles of periodization ^[1]^. Furthermore, growth and development phase of life has impact on training ^[2, 3]^. The training-induced changes observed in various physiological and biochemical parameters can be attributed to appropriate load dynamics.

Body composition has an important role in playing soccer ^[4, 5]^. Since in soccer lots of physical contacts occur and many movements and skills are involved a high level of physical demand is required which involves kicking, short sprinting, throwing, catching, trapping, etc ^[2, 3]^. Since soccer players have to cover a big area in the ground during attacks and defenses, the game demands for aerobic fitness as well as anaerobic one ^[3, 6]^. A high number of accelerations and decelerations associated with a large number of changes in direction of play create an additional load to the muscles involved. So, just those players who are suited to cope with these demands, reach elite levels ^[3, 7]^. The intermittent high intensity pattern of activity during matches requires a high function of both aerobic and anaerobic energy delivery pathways ^[8, 9]^. Moreover, power and strength have great impacts over the game which is required during sprinting and in execution of various skills with the ball ^[2, 3]^.

Oxygen is transported to muscles primarily by haemoglobin ^[10, 11]^. During aerobic exercise, the demand for oxygen increases at the working muscle; so an optimum level of hemoglobin is required to perform at the highest level with high intensity. As soccer performance depend mostly on aerobic component of the athlete, players need to maintain normal haemoglobin level to optimise their performance. Serum levels of urea and uric acid are sometimes used for assessment of training-related stress ^[12]^. During soccer training, these parameters may be evaluated at regular intervals to assess the training load imposed on athletes. In addition, urea and uric acid accumulation is most frequently used as a measure of protein catabolism and degradation of adenonucleotides ^[13–16]^. Lipids have important beneficial biological functions. These include usage of triglycerides for energy production, fat storage in adipose tissues, and usage of cholesterol as a component in phospholipids of cellular membranes or in the synthesis of steroid hormones ^[15, 17,18]^. Elevated plasma cholesterol concentrations have been implicated in the development of coronary artery disease (CAD)^[17, 19]^. Regular monitoring of these health-related variables of soccer players can provide valuable information about their health, metabolic and cardiovascular status.

This study has been focused on soccer players as this sport is the most popular one and played throughout the world. Physiological and biochemical variables have important role for evaluation of training and assessment of health, metabolism and cardiovascular status of soccer players. Regular monitoring of physiological and biochemical variables during training at various stages of growth and development may provide valuable information to coaches for training and selection of players at different age groups. Studies on the physiological and biochemical parameters of soccer players particularly in relation to training, growth and development are lacking in India. In view of the above, this study was designed to investigate the effect of a training program on selected physiological and biochemical variables of Indian soccer players of different age groups.

## METHODS AND SUBJECTS

**Subjects:** One hundred and twenty (N = 120) Indian male soccer players, regularly playing competitive soccer, volunteered for the present study. They were selected from the training camps at Sports Authority of India. The players were equally divided (n = 30) into 4 groups: under 16 years (U16, age: 14.00–15.99 yr, playing for last 2–3 years); under 19 years (U19, age: 16.00–18.99 yr, playing for last 4–7 years); under 23 years (U23, age: 19.00–22.99 yr, playing for last 8–11 years) and senior (SR, age: 23.00–30.00 yr, playing for last 12–17 years). Sportsmen under 16 years of age group were participating in different School levels and Sub-junior National level competitions. The players under 19 years of age group represented India in Junior World cup, Junior World Championship and Junior Asian Championship. Sportsmen under 23 years and senior age groups were participating in different International competitions including World cup, World Championship, Olympic Games and Asian Games.

**Training:** After taking the baseline data (BD, zero level) the players went through a training program. The training sessions were divided into two phases (i) Preparatory Phase (PP, 8 weeks), and (ii) Competitive Phase (CP, 4 weeks). The volume and intensities of the training components also varied in each phase of the training. In the preparatory phase, the volume and intensity of the training increased gradually. On the other hand, in the competitive phase the training volume and intensity was changed according to the competition schedule. At the same time, a highly specified training related to soccer and practicing for matches was followed in the competitive phase. The players generally completed an average of 2 hours of training in the morning sessions which was mostly performed to improve the physical fitness of the players. In the evening sessions, two hours of technical and tactical training was carried out which included dribbling, tackle, set up movements, penalty and match practices. The training sessions were followed 5 days/week, according to the requirements of the game and competitive demand. The training schedule, type of training, volume and intensity all are shown in table 1.

**Table 1:** General training schedule used for all the soccer players

Athletes name		Performance			Training objectives						Tactical preparation			Psychological preparation			
				Test/Standards	Physical preparation			Technical preparation									
**Competition type**		Domestic										x	x	x	x		**x**
**Periodization**		Training phase	Phase	Zero Level	Preparatory									Competitive			
			Sub-phase	Baseline	General preparation			Specific preparation						PC	Competition		
		Strength		–	AA			Maximal Strength						Power			
		Endurance		–	Aerobic			Anaerobic			Ergogenesis						
		Speed		–	Specific high			Specific									
		Skill Acquisition		–	Foundation			Advanced			Stimulation						
		Macro cycles		–	1			2						3		4	
		Micro cycles		–	1	2	3	4	5	6	7	8		9	10	11	12
**Peaking index**				–	4			4						3	2		
**Testing dates**				x									x				x
**Training factors**	**Volume**	100%	1	–	80–90%			70–90%						70%	60–70%		
	**Intensity**	90%	2	–	70–80%			80–90%						80%	80–90%		
	**Peaking**	80%	3	–	70–75%									80%	>90%		
	**Phys prep**	70%	4	–	50–55%			40–45%						30%	30%		
	**Tech prep**	60%	5	–	40–45%			40–45%						35%	35%		
	**Tact prep**	50%	Peaking	–	10%			10%						35%	35%		
	**Psych prep**	40%		–				10%						20%	30–35%		
		30%															
		20%															
		10%															

PC = Pre Competition, AA = Anatomical Adaptation.

**Testing:** The selected physiological and biochemical parameters were measured in the laboratory at the beginning of the training (baseline data, BD) and at the end of each training phase. Each test was scheduled at the same time of day (±1 hour) in order to minimize the effect of diurnal variation. All the experiments were performed at 25 ±1°C, with a relative humidity of 60–65%. The subjects were informed about the possible complications of the study and gave their consent.

**Fig. 1:**
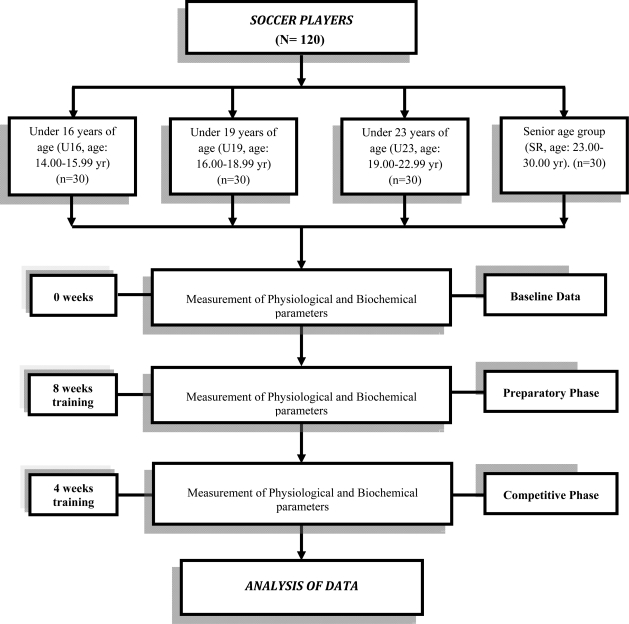
Schematic diagram showing the experimental design

Parental consent was also taken for the under 16-year age group players to participate in this study. The study was conducted at Sports Authority of India and was approved by the Human Ethical Committee of the Institute. The experimental design of the study is furnished in figure 1.

### Measurement of Physiological Variables

*Measurement of height (stature) and body mass:* The height was measured by the stadiometer (Seca 220, UK) with an accuracy recorded to the nearest 0.5 centimeters (cm). The subject stood barefoot, and erect with heels together and arms hanging naturally by the sides. The heels, buttocks, upper part of the back and usually but not necessarily, the back of the head were in contact with the vertical wall. The subject looked straight ahead and took a deep breath during measurement. The distance from the standing platform, to the highest position of head (vertex) was measured with the help of stadiometer, which indicates the subjects’ height ^[20]^. The stature was recorded in centimeters. The body mass was taken on a standard electronic weighing machine (Seca Alpha 770, UK), having an accuracy recorded to the nearest 50 grams (gm). The subject was examined in clothing of known weight in order to record nude weight 12 hours after the last meal. The subject stood at the centre of the weighing machine looking straight. The body mass was recorded in kilograms ^[20]^.

*Assessment of percent body fat and lean body mass:* A skin fold calliper (Holtain Limited, UK) was used to assess the body fat percentage following standard methodology ^[21]^. The instrument consists of accurately calibrated dial which indicates the thickness of the skin fold in millimetres (mm) when the skin fold is held by the open jaws. The skin fold was taken from four different sites of the body (biceps, triceps, sub-scapular and suprailiac) using the skin fold calliper on the right side of the body. The thickness of the skin and subcutaneous fat was grasped between the thumb and index finger. To estimate the errors, reading was made between three and four seconds when essentially all compressions have taken place and the measurements were established.

Computation of Body density (BD): Body density was calculated by the standard formulae ^[22]^. The skin fold thickness at the site of biceps, triceps, sub-scapular and suprailiac was used to calculate the body density.

Calculation for Body density (BD):

BD=1.1620−0.0630log(Biceps+Triceps+Sub scapular+Suprailliac)(for14−19yrs)

BD=1.1631−0.0630 log(Biceps+Triceps+Sub scapular+Suprailliac)(for20−29yrs)

BD=1.1422−0.0544 log(Biceps+Triceps+Sub scapular+Suprailliac)(for20−29yrs)

Computation of Percent Body fat was derived using the standard equation ^[16]^^.^

Body fat(%)=(495/Body density)−450

Computation of Lean body mass (LBM) was derived by subtracting fat mass from total body mass ^[20]^ using the following equation.

Fat mass(kg)=[Body mass(kg)×Body fat(%)]/100LBM(kg)=Body mass−Fat mass

*Determination of maximum oxygen consumption (VO_2max_) and heart rates:* The direct assessment of maximal aerobic capacity was achieved using a metabolic analyzer (Oxycon Champion, Jaeger, Germany) and a treadmill (Jaeger LE 500; Jaeger, Germany). The subjects were fitted with a face mask which was connected to an automated gas analyzer. This analyzer samples the expired air containing oxygen and carbon dioxide, and through a series of calculations, oxygen consumption is determined ^[23]^. The subject was instructed to avoid heavy food intake and exercise at least 2 hours before the treadmill test. The treadmill was attached to a computerized metabolic analyzer. The detailed procedure of the test was explained to the subjects and the demonstration of the test was shown to them. Subjects were given a trial run on treadmill at 0% gradient and at 4-km h^−1^, 5-km h^−1^, 6-km h^−1^, and 8-km h^−1^ for 2 minutes at each speed, with the face mask attached to the mouth-piece. Then the subject was asked to stand on the treadmill with the face mask attached to the mouth-piece, while the treadmill was stationary. Expired gases were sampled breadth-by-breadth and measured from a mixing chamber using a computerized metabolic analyzer. The heart rate, oxygen consumption, carbon dioxide production, pulmonary ventilation and respiratory quotient (RQ) were recorded while the subject was standing on the treadmill. The initial speed and the inclination of the treadmill were 8- km h^−1^ and 2%, respectively. The speed was increased by 2-km h^−1^ at every 2 minutes, until volitional exhaustion (RQ > 1.0). Oxygen consumption, heart rate, and respiratory quotient (RQ) were monitored for every 30 seconds. When the subject's heart rate leveled off, prior to the final exercise intensity at a value of at least 95% of the predicted maximum heart rate and his respiratory exchange ratio were greater than 1.0, the observed VO_2_ was considered as VO_2max_. During recovery, the treadmill speed was slowed down gradually and stopped and all the above physiological variables were monitored. The heart rate during exercise and recovery was also measured using Sport Tester (Polar, Finland). The Sports Tester can measure heart rate in 5 sec. intervals. The first minute recovery heart rate was recorded one minute after the cessation of exercise.

*Measurement of anaerobic power:* The Wingate Anaerobic Test (WANT) was performed using a cycle ergometer (Jaeger LE 900; Jaeger, Germany) following a standard methodology ^[24]^. The subject was instructed to avoid heavy food intake and exercise at least 2 hours before the test. The detailed procedure of the test was explained to the subjects and the demonstration of the test was shown to them. The subject was asked to follow the instructions of the investigator during the experiment. The subject was given a trial on cycle ergo meter. The test requires the subject to pedal a mechanically braked bicycle ergo meter for 30 seconds at an “all out” pace. The individual is advised to complete a warm-up for 3–5 minutes followed by a recovery cool down for 1–2 minutes. On commencing the test (usually by a verbal signal from the tester), the individual pedals “all out” with no resistance. Within 3 seconds, the predetermined fixed resistance of 0.075 kg per kg body mass was applied to the flywheel and the athlete continued to pedal “all out” for 30 seconds and the load remains throughout the test. A computerized counter was used to record alterations of the flywheel in 5-second intervals, although the actual test was performed for 30-second intervals. Anaerobic power was measured using the software supplied by Jaeger, Germany.

*Measurement of Back and grip Strength:* The back and grip dynamometers (Senoh, Japan) were used to record the strength of the back and grip muscles following a standard method ^[20]^. For measurement of back strength, one hand of the subject gripped over and the other under the bar. The hands were spread to the width of the shoulders. The trunk was flexed only slightly forward (10–15°) at the hip joints. The body weight was balanced on the feet which were placed about 15 cm. apart. The knees were kept straight throughout the lift. The lift was steadily upward, without jerking. The subjects were not allowed to lean backward on the heels. It was ensured that the back was almost straight at the end of the lift. For measurement of grip strength the dryness of the hand and the instrument were ensured. The tester set the pointer to zero and placed the dynamometer in the subject's hand, with the dial against the palm and the larger (concave) pressing edge in the “heel” of the palm. The posture and positioning of the subjects tested were according to a standard method ^[20]^. The data was obtained with the elbow at 90^o^ flexion, shoulder at 0^o^ flexion and wrist between 0° and 15° of ulnar and radial deviation. The subject squeezed sharply and steadily as much as possible, making certain that no part of the arm touched the body. For both back and grip strength test three trails were allowed with an interval of two minutes. The test was repeated in case any other deviation from proper procedure was noted. The highest reading of the three trials was recorded in kilogram.

**Measurement of Biochemical Variables:** Five (5) ml of venous blood was drawn from an antecubital vein after a 12-hour fast and 24 hours after the last bout of exercise for determination of hemoglobin, serum urea, serum uric acid, total cholesterol, triglycerol, HDL-C and LDL-C.

*Estimation of Hemoglobin:* Hemoglobin concentration was estimated using colorimetric procedure by Cyanmethaemoglobin method^[25]^. An aliquot of well-mixed whole blood was taken and reacted with a solution of potassium cyanide and potassium ferricyanide. The chemical reaction yields a product of stable color, the cyanmethaemoglobin. The intensity of the color is proportional to the haemoglobin concentration at 540 nm. The following reagents were used for the assay: (a) Reagent 1: Drabkin's Reagent (50 mg Potassium cyanide, 200 mg Potassium ferricianide and 1000 ml Distilled water); (b) Reagent 2: Cyanmathaeoglobin standard. All reagents were supplied by Merck Ltd., India. A three sets of test tubes were taken and marked as Blank, Test and Standard. In the Blank 5.0 ml of Reagent 1 was taken. The tube marked as Test contained 5.0 ml of Reagent 1, then 20 µl of an aliquot of well-mixed EDTA-anticoagulated blood specimen was added, mixed well and stand for 10 minutes. Another tube marked as Standard contained 5.0 ml of Cyanmathaeoglobin standard. Blank solution was used for setting the spectrophotometer. Absorbance (Abs.) of the Test and Standard was performed using spectrophotometer at 540 nm.

*Estimation of serum Urea:* Urea reacts with hot acidic Diacetylmonoxime in the presence of Thiosemicarbazide and produces a rose-purple coloured complex, which is determined colorimetrically^[26]^. The following regents were used for the assay: (a) Reagent 1: Urea Reagent; (b) Reagent 2: Diacetylmonoxime (DAM); (c) Reagent 3: Working Urea Standard, 30 mg%; Working solution was prepared with dilution of 1 ml of Reagent 1 to 5 ml with purified water (solution I). All reagents were supplied by Span Diagnostics Ltd., India. Three sets of test tubes were taken containing Standard (2.5 ml of solution I, 0.01 ml of Reagent 3 mixed well and 0.25 ml of Reagent 2 was added); Test (2.5 ml of solution I, 0.01 ml of serum sample mixed well and 0.25 ml of Reagent 2 was added) and Blank (2.5 ml of solution I and 0.25 ml of Reagent 2 was added). Then the samples were mixed well and test tubes were kept in the boiling water exactly for 10 minutes. After 10 minutes the test tubes were cooled under running water for 5 minutes. Measurement of the OD of Standard and Test was performed against Blank on a spectrophotometer at 525 nm within 10 min.

*Estimation of serum Uric acid:* Uric acid in alkaline medium reduces phosphotungstic acid to “tungsten blue” a blue coloured complex, which is measured colorimetrically^[27]^. The following regents were used for the assay: (a) Reagent 1: Sulphuric acid, 2/3 N; (b) Reagent 2: Sodium tungstate, 10% W/V; (c) Reagent 3: Sodium carbonate, 14% W/V; (d) Reagent 4: Phosphotungstate; (e) Reagent 5: Stock Uric acid standard, 100 mg%; Working Standard was prepared with dilution of 0.1 ml of stock Uric acid standard to 10 ml purified water and mixed well. All reagents were supplied by Span Diagnostics Ltd., India. The estimation of serum Uric acid was performed in two steps. Step I: Deproteinization of the serum sample was performed using 0.5 ml of serum, 4.0 ml of purified water, 0.25 ml of Reagent 1 and 0.25 ml of Reagent 2 taken in a test tube. The solutions were mixed well and stand for 10 minutes and then centrifuged at 2000 rpm for 15 minutes to obtain a clear supernatant. Step II: Three sets of test tubes were taken containing Standard (1.5 ml of working standard, 0.5 ml of Reagent 3 and 0.5 ml of Reagent 4); Test (1.5 ml of supernatant, 0.5 ml of Reagent 3 and 0.5 ml of Reagent 4) and Blank (1.5 ml of purified water, 0.5 ml of Reagent 3 and 0.5 ml of Reagent 4). Then the samples were mixed well and kept in darkness for 15 minutes. Measurement of the OD of Blank, Standard and Test was performed against purified water using a spectrophotometer at 710 nm.

*Estimation of serum total cholesterol (TC), high density lipoprotein cholesterol (HDL-C):* Cholesterol reacts with hot solution of Ferric Perchlorate, Ethyl Acetate and Sulphuric acid (Cholesterol Reagent) and gives a lavender coloured complex which is measured at 560 nm. High density lipoprotein cholesterol (HDL-C) is obtained in the supernatant after centrifugation. The Cholesterol in the HDL-C fraction is also estimated by this method ^[28]^. The following regents were used for the assay: (a) Reagent 1: Cholesterol Reagent; (b) Reagent 2: Working Cholesterol Standard, 200 mg%; (c) Reagent 3: Precipitating Reagent. All reagents were supplied by Span Diagnostics Ltd, India.

Estimation of serum Total cholesterol (TC): Three sets of test tubes were taken containing (i) Blank (3.0 ml of Reagent 1); (ii) Standard (3.0 ml of Reagent 1 and 15 µl of Reagent 2) and (iii) Test (3.0 ml Reagent 1 and 15 µl serum samples). Then the samples were mixed well and test tubes were kept in the boiling water bath exactly for 90 seconds (1½ minutes). Immediately after 90 seconds, the cooling of test tubes was done in room temperature under running tap water. Measurement of the OD of Standard and Test was performed against Blank on a spectrophotometer at 560 nm.

Estimation of serum High density lipoprotein cholesterol (HDL-C): Estimation of HDL-C was performed in two steps. The first step was the separation of HDL-C from total cholesterol in the serum samples. Secondly, estimation of HDL-C from the supernatant obtained from step one. Step I: A 0.2 ml of serum samples and 0.2 ml of precipitating reagent were taken in centrifuge tube. They were mixed well and kept at room temperature for 10 minutes and then centrifuged at 2000 rpm for 15 minutes to obtain a clear supernatant. Step II: Three sets of test tubes were taken (i) Blank (3.0 ml of Reagent 1); (ii) Standard (3.0 ml of Reagent 1 and 15 µl of Reagent 2) and (iii) Test (3.0 ml of Reagent 1 and 120 µl of supernatant from step 1). The samples were mixed well and the tubes were kept immediately in the boiling water bath exactly for 90 seconds (1½ minutes). Immediately after 90 seconds, the cooling of test tubes was done in room temperature under running tap water. Measurement of the OD of Standard and Test was performed against Blank on a spectrophotometer at 560nm.

*Estimation of serum Triglyceride (TG):* In the presence of enzyme *lipase,* triglycerides break into glycerol and fatty acids. Again, glycerol reacts with ATP and the reaction produces glycerol-3-phosphate and ADP. Enzyme *glycerolkinase* helps in this reaction process. Glycerol-3-phosphate reacts with oxygen in the presence of *glycerol-3-phosphate-oxidase* and produces dihydroxy-acetone-phosphate and hydrogen peroxide (H_2_O_2_). The hydrogen peroxide reacts with aminoantipyrine and chlorphenol in the presence of enzyme *peroxidase* and produces chinonimine and water ^[29]^. The following regents were used for the assay: (a) Reagent 1: Reaction solution 4×25 ml [Good's buffer (pH 7.2): 50 mmol l^−1^, 4-chlorphenol: 4 mmol l^−1^, ATP: 2 mmol l^−1^, Mg^2 + ^: 15 mmol l^−1^, glycerokinase: ≥0.4 KU l^−1^, peroxidase: ≥2.0 KU l^−1^, lipoproteinlipase: ≥2.0 KU l^−1^, 4-aminoantipyrine: 0.5 mmol l^−1^, glycerol-3-phosphate-oxidase: ≥1.5 KU l^−1^]; (b) Reagent 2: Standard solution 1×3 ml [triglycerides]. All reagents were supplied by Merck Ltd., India. Three sets of test tubes were taken containing Standard (1000 µl of reagent 1 and 10 µl of reagent 2); Test (1000 µl of reagent 1 and 10 µl of serum sample) and Blank (1000 µl of reagent 1). Then the samples were mixed well and incubate for 10 minutes at 37°C. Measurement of the OD of Standard and Test was performed against Blank on a spectrophotometer at 500 nm within 60 minutes.

*Assessment of low-density lipoprotein cholesterol (LDL-C):* Low-density lipoprotein cholesterol (LDL-C) was indirectly assessed using standard equation ^[30]^.

LDL−C=TC−(HDL−C+TG/5)

TC (total cholesterol); HDL-C (high density lipoprotein cholesterol), and TG (triglyceride), All values are in mg dl^−1^.

**Statistical Analysis:** All the values of physiological and biochemical parameters were expressed as Mean and Standard Deviation. Analysis of Variance (ANOVA) with repeated measures followed by multiple comparison tests was performed to find out the significant difference in selected physiological and biochemical parameters among the selected age categories; and within the different training phases. In each case the significance level was chosen at 0.05. SSPSS software for Windows was used.

## RESULTS

**Effect of training on body composition:** A significant (*P*<0.05) reduction in the percent body fat was reported among the soccer players of all age groups when comparing BD with that of the PP and CP. However, when comparing PP with that of the CP, no significant change was noted in the percent body fat (Table 2). On the other hand, a significant increase in lean body mass (LBM) was observed among the U19 age group players, but no significant difference in other age groups when comparing BD with that of the PP and CP (Table 2). Furthermore, no significant difference was observed in the body mass among the players of all age groups after the training program.

**Table 2:** Effect of training on body size and body fat of soccer players of different age groups

Parameters	Training phase	Age group (Yrs)			
		U16	U19	U23	SR
**Height (cm)**	**BD**	166.8±4.2	171.7±4.7	173.8±4.5	174.6±3.6
	**PP**	166.9±4.2	171.7±4.7	173.8±4.6	174.6±3.6
	**CP**	166.9±4.1	171.7±4.7	173.9±4.7	174.6±3.6
**Body mass (kg)**	**BD**	52.8±1.3	58.9±4.9	64.7±5.0	65.3±5.9
	**PP**	51.7±1.7	57.7±4.6	63.1±5.0	64.3±6.0
	**CP**	51.1±1.7	57.1±4.6	63.0±5.2	64.0±6.3
**Body fat (%)**	**BD**	16.6±2.0	14.3±2.3	13.9±2.2	12.7±2.7
	**PP**	15.0‡±2.7	13.4‡±2.9	12.1‡±2.2	12.0‡±2.6
	**CP**	15.0‡±2.1	13.0‡±1.3	12.1‡±1.5	12.0‡±2.2
**LBM (kg)**	**BD**	40.8±1.8	47.1±4.1	51.3±4.3	53.1±4.9
	**PP**	41.1±2.4	48.3^**‡**^±4.1	52.5±4.4	53.9±5.2
	**CP**	41.1±2.8	48.7^**‡**^±4.4	52.7±4.4	53.9±5.5

Data presented as mean ±SD; n = 30

Computed using alpha = 0.05

‡ when compared to BD, U16 = under 16 yrs, U19 = under 19 yrs, U23 = under 23 yrs, SR = senior age groups

BD = baseline data, PP = preparatory phase, CP = competitive phase

LBM = lean body mass

**Fig. 2:**
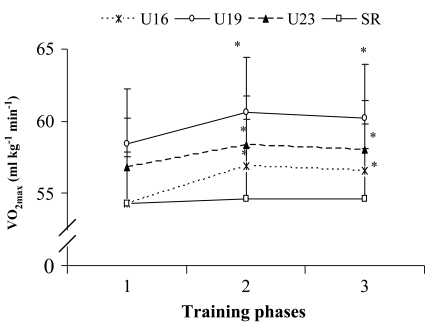
Effect of training on VO_2max_ of soccer players of different age groups Data presented as mean and SD, n = 30; *P*<0.05; Data were significantly different from each other when compared to (1 *P<0.050), 2 #*P*<0.050); U16 = under 16 yrs, U19 = under 19 yrs, U23 = under 23 yrs, SR = senior age groups; 1 = baseline data, 2 = preparatory phase, 3 = competitive phase; VO_2max_=maximal aerobic capacity.

**Effect of training on VO**_**2max**_ **and heart rate:** A significant increase (*P*<0.05) in relative VO_2max_ was noted in U16, U19 and U23 age groups in the PP and CP when compared to BD. However, no significant change was noted in relative VO_2max_ values in senior soccer players after the training program. In addition, when comparing PP with that of the CP, no significant change occurred in relative VO_2max_ value of the players of any age group (Fig. 2). No significant change was noted in maximal heart rate (HRmax) among the soccer players of any age group after the training. On the other hand, when comparing BD with that of the PP and CP, a significant reduction (*P*<0.05) in recovery heart rate was noted among the players of U16, U19, U23 and senior age group players. However, no significance difference in recovery heart rate was noted when comparing PP with that of the CP (Table 3).

**Table 3:** Effect of training on heart rates of soccer players of different age groups

Parameters	Training phase	Age group (Yrs)			
		U16	U19	U23	SR
**HRmax (beats min**^**−1**^**)**	**BD**	193.8±3.7	189.5±3.5	185.5±3.4	181.7±4.6
	**PP**	193.2±5.6	188.0±4.9	184.4±5.6	181.3±5.4
	**CP**	193.3±5.1	188.1±5.7	184.0±5.5	181.0±4.1
**RHR1 (beats min**^**−1**^**)**	**BD**	162.7±4.8	155.8±4.6	154.9±3.6	151.6±3.3
	**PP**	159.3‡±4.9	151.9‡±3.8	150.3‡±4.4	148.7‡±4.7
	**CP**	160.3‡±4.9	151.9‡±4.3	150.6‡±4.2	149.2‡±4.7

Data presented as mean ±SD; n = 30;

Computed using alpha = 0.05

‡ when compared to TP, U16 = under 16 yrs, U19 = under 19 yrs, U23 = under 23 yrs, SR = senior age groups

HRmax = maximal heart rate, RHR1 = Recovery heart rate 1^st^ min, BD = baseline data, PP = preparatory phase, CP = competitive phase.

**Fig. 3:**
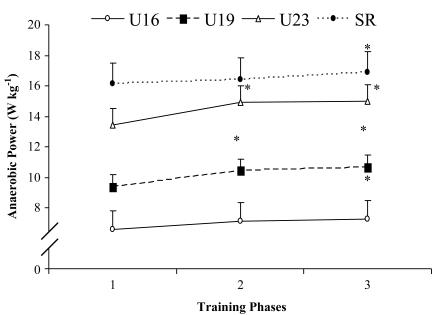
Effect of training on anaerobic power of soccer players of different age groups Data presented as mean and SD, n = 30; *P*<0.05; Data were significantly different from each other when compared to (1 *P<0.050, 2 #P < 0.050); U16 = under 16 yrs, U19 = under 19 yrs, U23 = under 23 yrs, SR = senior age groups; 1 = baseline data, 2 = preparatory phase, 3 = competitive phase

**Effect of training on anaerobic power and strength:** Anaerobic power (AP) increased significantly (*P*<0.05) among the U19 and U23 age group players but not in the U16 and senior age group players when comparing BD with that of the PP. However, when comparing BD with that of the CP, a significant (*P*<0.05) increase in AP was detected among all age groups. In addition, when comparing PP with that of the CP, no significant change was noted in AP of the soccer players of any age group (Fig. 3). The back and grip (right and left hand) strength increased significantly (*P*<0.05) among the soccer players of all age groups when comparing BD with that of the PP and CP. However, when comparing PP with that of the CP, no significant change observed in back and grip strength values of the players of any age group (Table 4).

**Table 4:** Effect of training on back and grip strength of soccer players of different age groups

Parameters	Training phase	Age group			
		U16	U19	U23	SR
**Back strength (kg)**	**BD**	105.7±4.2	112.2±4.7	118.9±3.1	124.2±4.6
	**PP**	107.0‡±4.2	114.5‡±4.5	122.5‡±4.5	125.4‡±4.5
	**CP**	107.8‡±4.3	114.6‡±4.9	123.3‡±4.9	125.8‡±3.0
**Grip strength of right hand (kg)**	**BD**	27.6±3.3	31.2±3.8	36.2±3.4	39.8±3.5
	**PP**	29.0‡±3.9	33.1‡±3.4	39.9‡±3.5	40.0‡±3.8
	**CP**	29.1‡±3.7	33.3‡±3.2	37.8‡±4.0	40.5‡±3.3
**Grip strength of left hand (kg)**	**BD**	26.6±3.9	30.5±2.8	33.9±3.6	35.3±3.5
	**PP**	29.3‡±3.2	32.2‡±3.8	35.5‡±3.7	36.8‡±3.4
	**CP**	29.5‡±3.2	32.6‡±3.2	35.9‡±3.9	36.6‡±3.3

Data presented as mean ±SD; n = 30

Computed using alpha = 0.05

‡ when compared to TP, U16 = under 16 yrs, U19 = under 19 yrs, U23 = under 23 yrs, SR = senior age groups

BD = baseline data, PP = preparatory phase, CP = competitive phase.

**Effect of training on hemoglobin, serum urea and serum uric acid levels:** A significant (*P*<0.05) reduction in the hemoglobin level was reported among the U19 and senior age group players, but not in U16 and U23 age groups when comparing BD with that of the PP. However, when comparing BD with that of the CP, a significant (*P*<0.05) reduction in the hemoglobin levels was observed among U19, U23 and senior age group players, with no significant change in U16. In addition, when comparing PP with that of the CP, no significant change was noted in the hemoglobin levels of any age group (Table 5). Serum urea level increased significantly (*P*<0.05) among all groups when comparing BD with that of the PP and CP. Moreover, when comparing PP with that of the CP, a significant (*P*<0.05) increase in the serum urea level was reported among the players of U19 and U23 age groups, but not in U16 and senior age groups (Table 5). When comparing BD with PP, no significant change in the serum uric acid level was noted among all age groups. However, when comparing BD with that of the CP, a significant (*P*<0.05) increase in the serum uric acid level was reported among the players of U19, U23 and senior age group, but not in U16 age group. In addition, when comparing PP with CP, no significant change in the serum uric acid level occurred among the soccer players of any age group (Table 5).

**Table 5:** Effect of training on haemoglobin, urea and uric acid levels of soccer players of different age groups

Parameters	Training phase	**Age group**			
		U16	U19	U23	SR
**Haemoglobin (gm/dl**^**−1**^**)**	**BD**	13.7±0.5	14.0±0.6	14.4±0.5	14.5±0.6
	**PP**	13.5±0.6	13.6‡±0.6	14.1±0.8	14.3‡±0.7
	**CP**	13.4±0.6	13.6‡±0.6	14.0‡±0.7	14.1‡±0.6
**Urea (mg/dl**^**−1**^**)**	**BD**	22.7±2.0	27.1±2.7	28.1±2.8	29.3±2.3
	**PP**	23.1‡±2.0	28.0‡±3.0	29.2‡±2.3	30.4‡±2.5
	**CP**	27.8‡±2.7	31.3‡†±2.9	32.1‡†±2.5	32.1‡±2.2
**Uric acid (mg/dl**^**−1**^**)**	**BD**	3.1±0.3	3.5±0.5	4.2±0.6	4.1±0.5
	**PP**	3.4±0.2	3.9±0.5	4.5±0.6	4.3±0.5
	**CP**	3.7±0.2	3.9‡±0.6	4.8‡±0.6	4.6‡±0.5

Data presented as mean ±SD; n = 30

Computed using alpha = 0.05

‡ when compared to TP

† when compared to PP; U16 = under 16 yrs, U19 = under 19 yrs, U23 = under 23 yrs, SR = senior age groups

BD = baseline data, PP = preparatory phase, CP = competitive phase.

**Fig. 4:**
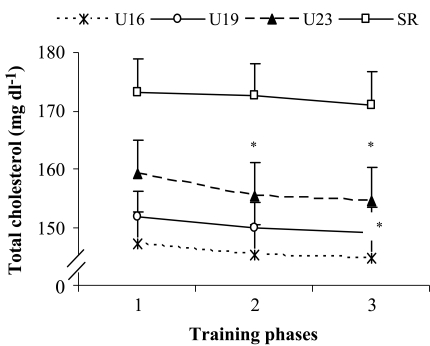
Effect of training on total cholesterol of soccer players of different age groups Data presented as mean and SD, n = 30; *P*<0.05; Data were significantly different from each other when compared to (1 *P<0.050, 2 #*P*<0.050); U16 = under 16 yrs, U19 = under 19 yrs, U23 = under 23 yrs, SR = senior age groups; 1 = baseline data, 2 = preparatory phase, 3 = competitive phase

**Effect of training on total cholesterol, triglyceride, HDL-C and LDL-C levels:** A significant (*P*<0.05) reduction in total cholesterol (TC) level was detected only in U23 age group, but not in U16, U19 and senior age groups when comparing BD with that of the PP. In addition, when comparing BD with CP, a significant (*P*<0.05) reduction in TC level was reported among U19 and U23 age groups, but not in U16 and senior age groups. However, when comparing PP with CP, no significant change in TC level was observed among the players irrespective of age groups (Fig. 4). The triglyceride (TG) level reduced significantly (*P*<0.05) among U23 age group, but not in U16, U19 and senior age groups when comparing BD with that of the PP. Furthermore, when comparing BD with CP, a significant (*P*<0.05) reduction in TG level was reported among U23 and senior age groups, but no significant change was noted in U16 and U19 age groups. In addition, when comparing PP with that of the CP no significant change in TG level was observed among the players of any age group (Table 6). In addition, a significant (*P*<0.05) reduction in LDL-C level was reported among U19 age group, but not in U16, U23 and senior age groups when comparing BD with that of the PP and CP. In addition, when comparing PP with that of the CP, no significant change in LDL-C level was observed among the players of any age groups (Table 6).

**Table 6:** Effect of training on triglyceride and LDL-C levels of soccer players of different age groups

Parameters	Training phase	Age group			
		U16	U19	U23	SR
**Triglyceride (mg dl**^**−1**^**)**	**BD**	58.6±4.3	64.9±5.5	87.9±5.2	104.1±6.1
	**PP**	56.4±4.9	62.7±5.7	85.1‡±5.7	102.4±6.2
	**CP**	55.8±5.2	62.2±5.7	84.7‡±5.1	101.3‡±6.8
**LDL-C (mg dl**^**−1**^**)**	**BD**	99.4±5.1	102.4±5.3	100.4±4.1	105.5±5.6
	**PP**	97.6±5.4	101.0‡±4.8	99.0±5.4	103.4±5.4
	**CP**	96.7±5.3	100.0‡±4.3	98.2±5.9	103.1±5.2

Data presented as mean ±SD; n = 30

Computed using alpha = 0.05

‡ when compared to TP, † when compared to PP

U16 = under 16 yrs, U19 = under 19 yrs, U23 = under 23 yrs, SR = senior age groups

BD = baseline data, PP = preparatory phase, CP = competitive phase

LDL-C = low density lipoprotein cholesterol.

**Fig. 5:**
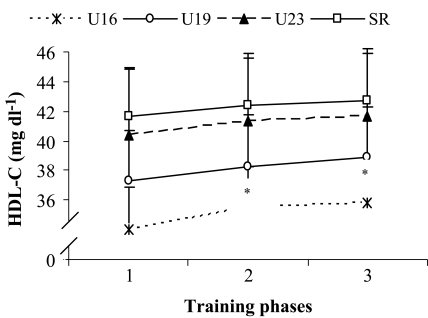
Effect of training on HDL-C level of soccer players of different age groups Data presented as mean and SD, n = 30; P < 0.05; Data were significantly different from each other when compared to (1 *P<0.050, 2 #P < 0.050); U16 = under 16 yrs, U19 = under 19 yrs, U23 = under 23 yrs, SR = senior age groups; 1 = baseline data, 2 = preparatory phase, 3 = competitive phase; HDL-C = high density lipoprotein cholesterol

HDL-C level increased significantly (*P*<0.05) among the players of U16 age group, but not in U19, U23 and senior age groups when comparing BD with that of the PP and CP. However, when comparing PP with that of the CP, no significant change in HDL-C level was observed among any age group (Fig. 5).

## DISCUSSION

Body size (height and body mass) has a significant impact on soccer players ^[4, 31,32]^. The tall players are recruited as goal keepers, defenders and forward positions; however a standard height should be maintained for midfield players. Body mass is a considerable factor in soccer since body contact is essential in this game ^[2, 33]^. In this study no significant difference was observed in body mass among the soccer players after the training program. It may be due to the short duration of the training or improper optimization of the training load. It has been reported that short term exercise training has no significant effect on body mass of sportsmen ^[34, 35]^.

The percentage of body fat plays an important role for the assessment of physical fitness of soccer players^[2, 36,37]^. Generally, the amount of fat in an adult male in his mid-twenties is about 16.5% of body weight ^[3, 34,35]^. A lean body is desirable for sports like soccer ^[2, 36,37]^. A low-body fat may improve athletic performance by improving the strength-to-weight ratio ^[3, 34,35]^. Excess body fat adds to the load without contributing to the body's force-producing capacity ^[3, 34,35]^. A reduction (*P*<0.05) in percent body fat was noted among the players after the training. The possible reason of reduction of body fat is exercise training which increases greater utilization of fat for energy ^[34, 35]^. However, significant increase in LBM was noted only in U19 age group, but other groups showed no significant differences in LBM after the training. This may be due to short duration of the training program. Similar findings were also noted by other research groups who studied on soccer players and reported that percent body fat decreased significantly during preparatory and competitive phases of training when compared to baseline data ^[3, 38]^. Therefore, it can be stated that soccer players can accumulate body fat in the off seasons when there is no training, and lose body fat during preparatory and competitive phases of training ^[34, 35]^. This might be due to intensive training during preparatory phase and high levels of performance during competitive phase ^[34, 35]^. Before and after competition season, during the interval periods, fat content of most soccer players increase presumably as a result of reduced aerobic activity along with nutritional and behavioral changes ^[34, 35]^. It is advised to perform low intensity aerobic endurance exercises during the off seasons in order to reduce the excess accumulation of body fat during the off seasons.

The maximal oxygen uptake (VO_2max_) is the best overall measure of aerobic power ^[3, 6]^. Aerobic capacity certainly plays an important role in soccer and has a major influence on technical performance and tactical choices ^[3, 34,35]^. An increase (*P*<0.05) in relative VO_2max_ value was found noted in U16, U19 and U23 age groups in the preparatory and competitive phases when compared with that of the baseline data, but not in senior age group. In U16, U19 and U23 the increase in VO_2max_ after training may be due to an increase in the systemic a-v O2 difference and stroke volume, when compared to senior players ^[34, 35]^. Moreover, these changes may be the result of increased volume of endurance training in preparatory phase ^[34, 35]^. The aerobic endurance training enhances the activity of the cardiovascular system as well as developed oxidative capacity of the skeletal muscles which leads to an increase in the delivery of oxygen to working muscles ^[34, 35]^. This is accepted as the main reason for elevation of VO_2max_ after a training program ^[34, 35]^. Furthermore, a decline in VO_2max_ values was observed, although non-significantly, from preparatory phase to competitive phase. This may be because of the reduction in aerobic training during competitive phase. On the other hand, no significant change was noted in VO_2max_ values of the senior age group after the training program in preparatory and competitive phases when compared with the baseline data. It shows that VO_2max_ of the soccer players may improve with training and the VO_2max_ of the U16, U19 and U23 age groups improves significantly in comparison to the senior players. Similar observation has been reported previously ^[2, 3,7]^. The extent by which VO_2max_ could been changed with training also depends on the starting point ^[34, 35]^. The fitter an individual is to begin with, the less potential there is for an increase and most elite athletes hit this peak early in their career ^[34, 35]^. There also seems to be a genetic upper limit beyond which further increases in either intensity or volume have no effect on aerobic power ^[34, 35]^. Other than tactical and technical aspects of soccer, monitoring of VO_2max_ is essential during the training phases, which helps the coaches for selection of players for competition.

Heart rate increases with an increase in work intensity and shows a linear relationship with work rate ^[23]^. The highest rate at which the heart can beat is the maximal heart rate (HRmax). Quick recovery from strenuous exercise is important in soccer which involves intermittent efforts interspersed with short rests ^[34, 35,39]^. The heart rate recovery curve is an excellent tool for tracking a person's progress during a training program ^[34, 35]^. A significant decrease (*P*<0.05) in recovery heart rate was observed among all age groups after the training program used here. Exercise cardio acceleration results from release of parasympathetic inhibition at low exercise intensities and from both parasympathetic inhibition and sympathetic activation at moderate intensities ^[34, 35]^. Nevertheless, parasympathetic activation is considered to be the main mechanism underlying exponential cardio deceleration after exercise ^[34, 35]^. On the other hand, no significant change was detected in HRmax of the players after the training course. This may be due to short duration of the training which has been shown in former studies ^[34, 35]^. The results of the present study suggest that the strain on the circulatory system during playing soccer is relatively high. Exercising at this intensity should provide a good training stimulus. Therefore, heart rate monitoring is essential during the training seasons, which also provides a database to the coaches for selection of players.

Soccer demands high anaerobic power as quick acceleration and deceleration are important in this sport ^[2, 3]^. Although most time of the game is spent in low-level activities such as walking and light jogging, repeated back-to-back sprints make speed and tolerance to lactic acid an important characteristic in players ^[2, 3]^. A high anaerobic power is essential for such activities ^[2, 3]^. Thus a high anaerobic power helps to develop sprint quality of the players ^[2, 3]^. Anaerobic power represents the highest rate of anaerobic energy released ^[34, 35]^. On the other hand, strength is the central component of a soccer training program ^[2, 3,37]^. Strength of the back muscles plays a key role of fitness among the soccer players, as kicking, passing, changing pace etc. are part of the game ^[2, 3]^. Therefore, the game demands high level of back strength. On the other hand, strength of grip muscle also has significant impacts on the performance of soccer players, which is needed for throw-in, catching or fisting the ball (goal keeping) ^[2, 3]^. A significant increase in anaerobic power and strength took place after the training among the soccer players. The greater gain in anaerobic power and strength was observed in U16 and U19 age groups, but less gain was noted in U23 and senior age group players. This may be due to maturation factors and/or motivation. However, the less gain in relative anaerobic power and strength output in senior players may be due to age decline. Similar observation has been observed by many researchers ^[2, 3,37, 40]^. They studied on soccer players and reported that the strength and power increased after training ^[2, 3,37]^. Monitoring of power and strength at regular intervals is essential during the training seasons, which helps in selection of players for competitions.

Oxidative potentiality of an athlete is dependent on his hemoglobin level ^[34, 35]^. Increase in VO_2max_ demands higher rates of oxygen supply ^[34, 35]^. Oxygen is transported to muscle primarily by hemoglobin (Hb), and it is suggested that hemoglobin mass and/or concentration is related to VO_2max_^[16]^. Then the training load was increased in preparatory and competitive phases form the zero level. Therefore in preparatory and competitive phases, reduced (*P*<0.05) hemoglobin level was observed compared to baseline data. Moreover, as the performance level increased in the competitive phase, the decline in hemoglobin level became more prominent when compared with the transition phase. Similar observations have been noted by many researchers in their recent studies ^[11, 16]^. The decline in hemoglobin level may be due to haemolysis ^[11, 41]^. Intravascular haemolysis is one of the most emphasized mechanisms for destruction of erythrocytes during and after physical activities ^[42, 43]^. In addition, exercise training-induced reduction in hemoglobin concentration also may be due to hemodilution which is a common physiological effect of endurance training which exists among the well trained athletes due to increased in plasma volume ^[16, 44]^. A recent study reported declined haematocrit during the race and continued falling on the next day with a corresponding rises in plasma volume following an ultra endurance cycling. They reported that the impact on the plasma volume is pronounced leading to marked haemodilution post-exercise ^[44]^. Another study reported a decrease in hemoglobin concentration during the post-race recovery period following an ultra marathon race. The greatest reduction in hemoglobin concentration was observed 48 hours after the race ^[45]^. The authors suggested that this reduction in hemoglobin concentration was due to hemodilution ^[45]^. Since hemoglobin level has relation with VO_2max_, an optimum level of hemoglobin is required for better performance. In view of the above, monitoring of hemoglobin level during the training phases is essential.

The present study showed that the level of serum urea and uric acid increased (*P*<0.05) after training among the players irrespective of age groups. However, the urea and uric acid levels were found to be in the normal range. The highest serum levels of urea and uric acid were found to be in the competitive phase when the performance level is the highest. It is believed that a pronounced increase in the urea and uric acid concentrations indicates strong influence of a training session, whereas normalization of the urea and uric acid level in blood is an index of time to perform subsequent strenuous training sessions ^[12]^.

A recent study suggested that serum uric acid scavenges OH_2_ radicals and there is evidence that it may be an important biological scavenger against free radicals in human plasma and in skeletal muscle during and after acute hard exercises ^[46]^. It has been reported by some researchers that during intensive exercise OH_2_ radicals are produced which can damage the cell membrane ^[46]^. It can be stated that as the OH_2_ radicals are produced during exercise, the serum level of uric acid also elevates to scavenge them. Regular monitoring of serum urea and uric acid levels can indicates strong influence of a training session, whereas normalization of the urea and uric acid level in blood is an index of time to perform subsequent strenuous training sessions. Therefore, these parameters may be used to assess the training load imposed on the players.

Lipids and lipoprotein profiles indicate the cardiovascular and metabolic status of athletes ^[17, 47]^. Activity levels have significant impacts on the lipids and lipoprotein levels of athletes ^[17]^. As the performance level increased during the preparatory phase and further to competitive phase, the level of total cholesterol, triglyceride and LDL-C decreased (*P*<0.05), and the level of HDL-C increased (*P*<0.05) gradually. It indicates that as the training load and performance level increase the level of total cholesterol, triglyceride and LDL-C decrease gradually with an increase in HDL-C level. The possible reason for the reduction in total cholesterol, triglyceride and LDL-C, and elevation in HDL-C is exercise training ^[15, 17,35]^. Our findings are supported by observations of other researchers in their recent studies ^[15, 17]^. Cross-sectional studies also reported an increase in HDL-C level and decrease in triglyceride level after exercise ^[17, 15,48, 49]^. A recent study showed significant increase in HDL-C level and decrease in LDL-C level, with no change in triglyceride after 9 weeks of training ^[14]^. Another study reported that 4 weeks of aerobic exercise training significantly decreased the levels of total cholesterol, LDL-C, and increased HDL-C ^[18]^. Therefore, regular monitoring of lipids and lipoproteins profiles of soccer players is essential to optimize their health status which has direct effect on performance of the players.

## CONCLUSION

Training effects were reflected on various parameters like body fat, aerobic capacity, anaerobic power, strength, haemoglobin, urea, uric acid, and lipid profile of Indian soccer players of different age groups. The unique profile of age-related changes should be taken into consideration while administering training to the players. Since studies on soccer players are limited in India, the data of the present study can be a handy tool and can act as a frame of reference for monitoring the training courses of soccer players of different age groups. This would enable coaches to assess the current status of an athlete and the degree of training adaptability and to provide an opportunity to modify the training schedule accordingly to achieve the desired performance. The effect of training can also be studied at different micro cycles to observe the effect of a particular training goal on the players. The study was restricted to soccer players, however similar studies can be performed in other sports disciplines. Selected physiological and biochemical variables were considered in the present study, however with the advancement of science and technology some more variables may be considered as well as new equipments can be used for assessment of variables.
